# Prevalence of middle mesial canal in mandibular molars: A cross-sectional cone-beam computed tomography study

**DOI:** 10.4317/jced.61844

**Published:** 2024-09-01

**Authors:** Marta Bascón-Mallado, Alberto Cabrera-Fernández, Daniel Cabanillas-Balsera, Isabel Crespo-Gallardo, Manuel Pabón-Carrasco, Jenifer Martín-González

**Affiliations:** 1 Department of Stomatology, Endodontic Section, School of Dentistry, University of Sevilla, Sevilla, Spain; 2 Department of Nursing, School of Nursing, University of Sevilla, Sevilla, Spain

## Abstract

**Background:**

The first tooth to erupt is the first mandibular molar, which is the tooth with the highest number of retreatments. Several factors are responsible for the failure of the endodontic success and one of the most important being the particular pulp anatomy of each tooth. To aim was determine the prevalence of the middle mesial (MM) canal in first mandibular molars and to study if there are predisposing factors to the presence of this canal by retrospectively analyzing cone-beam computed tomography (CBCT) images in vivo.

**Material and Methods:**

CBCT images of 100 patients were selected. A total of 206 first mandibular molars were examined. The CBCTs were analysed using Careastream CS 3D imaging software. Findings of MM canals were recorded along the variables sex and left/right side. Prevalence was compared using the Chi-square test (*P*< 0.05) and Cramer’s V was used to indicate the effect size between the variables.

**Results:**

Of the 206 teeth analysed, the prevalence of MM canals was 33.11% (49 teeth). There was no statistically significant difference between sex and prevalence of the MM canal. There was a significant difference between sex and the distance between the mesial canals was found, being the most common range in women was 1-2 mm (35.8%) and 2-3 mm (51%) in men. The most common range of distance between the mesial canals where the MM canal was localized was 3-4 mm (50%), being statistically significant (*p*<0,05).

**Conclusions:**

This cross-sectional study showed a high prevalence of MM canals (33.11%) in first mandibular molars. The prevalence of the MM canal was significantly higher when the distance between mesiobucal and mesiolingual canals was 3-4mm. This knowledge let direct the clinicians in locating MM canal for improving endodontic prognosis.

** Key words:**Cone-beam computed tomography, middle mesial canal, prevalence.

## Introduction

Mandibular molars are essential teeth to support the occlusal load of chewing (1). Both teeth frequently suffer the consequences of caries, and develop irreversible pulpitis, pulp necrosis and/or apical periodontitis, which implies the need of root canal treatment (RCT).

The number of root canals most frequently found in the mesial root of both mandibular molars is two, while in the distal root it is most common to find only one root canal (2). Numerous studies have examined the internal morphology of mandibular molars using to describe it the Vertucci’s conFigurations (3). Pérez Heredia *et al*. found that 94% of the first mandibular molar and 83% of the second mandibular molars had two roots, the first mandibular molar being the only one in which, in a very low percentage, three roots were found. Moreover, they found the most frequent Vertucci’s conFigurations were types II and IV for mesial roots and type I for distal roots of both molar teeth (4). Gambarini *et al*. (2018) found the same results for Vertucci’s conFiguration, being the most frequent type II and IV for the mesial roots (5). However, other studies have found that Vertucci’s type IV conFiguration was the most common at the mesial roots, and type I conFiguration at the distal roots for the mandibular first and second molars (6,7).

The variations in the anatomy of mandibular molars are associated to ethnicity (4), such as the presence of a distolingual root or radix entomolaris in Mongolian breeds, and the presence of a C-duct in uniradicular molars (8)(6). One of the anatomical variations that can be observed in mandibular molars is the presence of a middle mesial canal (MMC) in the mesial root. There was a great variation in its prevalence, from 14,7% (9), to 59% (10), passing by 3,13% (11), 3,41% (12) and 2,6% (13). The incorporation of the CBCT to endodontic practice has demonstrated that the presence of the MMC in the mesial root of mandibular molars reaches frequencies from 15% - 46% (9,14-16).

Taking into account that knowledge of this anatomical variation in the mandibular first and second molars is key to successful RCT, and considering that there are available limited data (4) about the prevalence of MMC in the mandibular molars in Spain, the null hyptothesis is that there is no relationship between the MMC with other anatomic factors. The main objective of this study was to determine the prevalence of the MMC. The secondary objective was to determine any correlation between the MMC with variables such as sex, distances between mesial canals, distances between mesial canals to the vestibular cortical bone.

## Material and Methods

This is a cross-sectional study conducted during 2024. The study was approved by the Research Ethics Committee of the University of Seville. The Strengthening the Reporting of Observational Studies in Epidemology (STROBE) guidelines was used to design the study protocol.

-Study sample

One hundred of CBCT images, corresponding to 100 patients treated in the Master of Endodontics at the University of Seville, acquired randomly during 2024, were included in the investigation. All patients had signed informed consent and authorization so that their CBCT could be used in research.

The American Association of Endodontist /American Academy of Oral and Maxillofacial Radiology Position on Cone Beam Computed Tomography guideline were considered for the adquisition of CBCT imaging (17).

All of the patients came to the Master´s course as a result of referrals from the undergraduate and other Master’s courses at the Faculty, not because of complex anatomy, but because of the need for endodontic treatment.

Inclusion criteria were as follows: 1) CBCT with a resolution of 90 microns or less; 2) showing fully erupted first mandibular permanent molars with mature apex. Exclusion criteria were: first mandibular molars with open apices, root resorption, calcifications, root canal treatments, posts, crowns, developmental disorders, pathologies, or history of orthodontic treatment. For each CBCT, the sex and age of the patient were registered.

-Imaging method

All the CBCT images included in this study was acquired using a Carestream 8100 3D unit (Carestream Dental LLC, Atlanta, USA). It had a tube voltage of 90 kVp, a tube current of 4 mA (pulse mode), and a field of view of 75×75×75 micrometers.

Some examples of the diagnostic CBCT images were included in the study.

-CBCT Analysis

CBCT images were examined using a Careastream CS 3D imaging software in an Intel Core i7–4460 at 3.20GHz PC workstation (Intel Corp, Santa Clara, CA, USA), running Windows XP professional SP-2 (Microsoft Corp, Redmond, WA, USA). All the CBCT images were examined by two observers (one endodontist and one general practitioner), who evaluated the presence of the MMC in mandibular first molars.

In order to calibrate the two observers, an endodontist with more than 30 years of clinical experience provided instructions about the protocol to be followed to them. This protocol was a step-by-step with respect the anatomical landmark definitions and to the CBCT scanning methodology. All the question and doubts were solved by the oldest endodontist.

Nevertheless, the degree of agreement was very high between the two assessors. The concordance between the two evaluators was estimated to be very high (kappa index 0.97).

**Figure 1:**
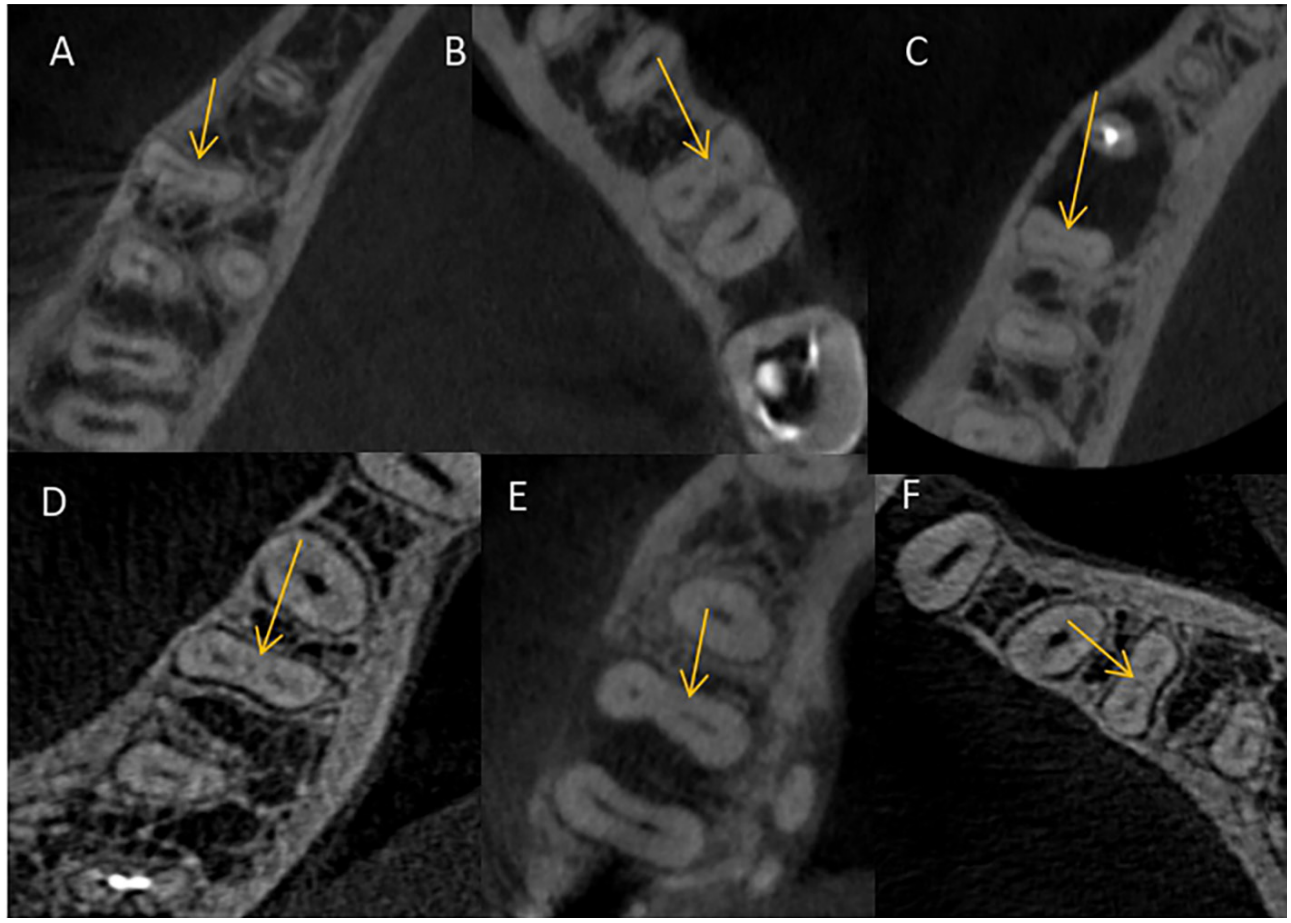
Axial view of the mesiocentral canals.

**Figure 2:**
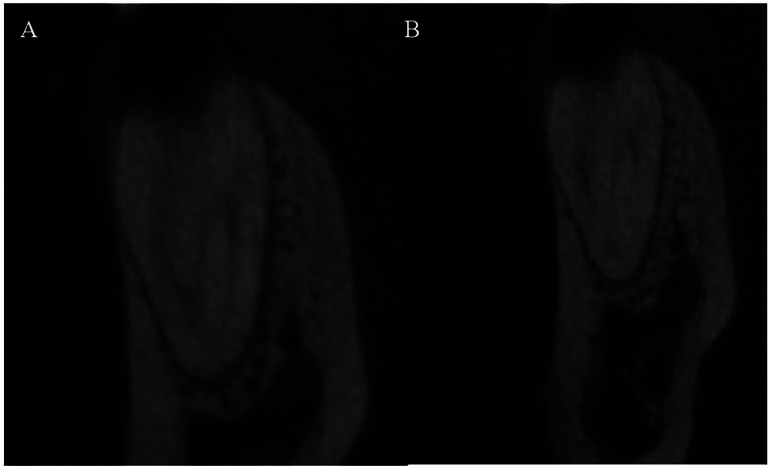
Coronal view of the mesiocentral canal.

All mandibular first molars were thoroughly examined in the three planes (axial, sagittal, and coronal) at 1.0 mm intervals by continuously moving the toolbar from the floor of the pulp chamber to the apex. It was evaluated the presence of the MMC according to the classification of Pomeranz *et al*. (18). In both axial and coronal view, the MMC was recorded as a visible when: 1) a radiolucency with a distinct round cross section began in the coronal third independent of the mesiolingual (ML) or mesiobuccal (MB) root canals, 2) fin, 3) fused with the MB root canals, 4) fused with the ML (Figs. 1,2).

**Figure 3:**
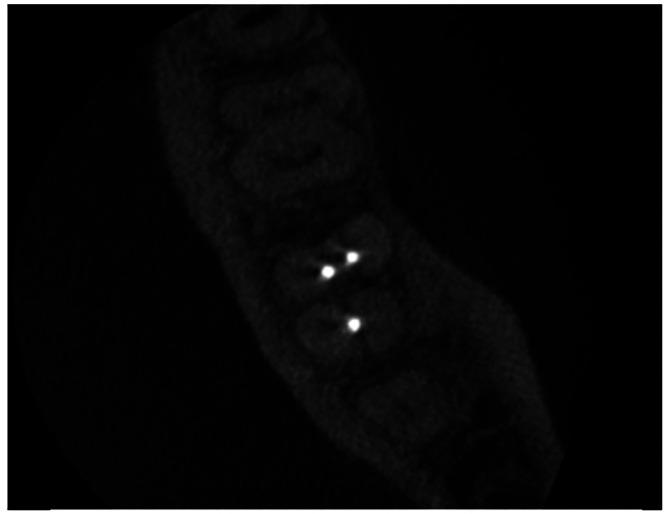
Distances between mesial canals.

Moreover, the distance between the mesial canals was recorded in the horizontal section, positioning the section at the level of the pulp chamber, and lines were drawn from the closest proximal surface between the analysed canals (Fig. 3). The conFiguration of the root canal anatomy of the MMC was compiled according to Vertucci’s conFiguration (3).

-Statistical Analysis

The data obtained were recorded in an Excel spreadsheet. Data analysis was performed with the help of the Statistical Package for Social Sciences Version 24 (SPSS Inc., Chicago, IL, USA). Frequencies and percentages were provided for qualitative variables as well as the mean and standard deviation together with their 95% confidence intervals for quantitative variables. Differences in the prevalence of the MMC based on sex and on distance between ML and MB canals were compared using the Chi-square test with a level of significance as *P* < 0.05. The effect size was estimated using Cramer’s V-test (0.1 to 0.3: Weak association; 0.3 to 0.5: Moderate association; 0.5 to 0.7: Strong association; 0.7 to 1: Very strong association).

The sample calculation was performed using a power of 0.90 for an error of 0.05 and a mean effect size of 0.4 (test method: X2, G* Power 3.0.10, Franz Faul, University of Kiel, Kiel, Germany). A minimum of 89 CBCTs was estimated to be necessary. This was extended by 15% to allow for possible losses. A total of 102 CBCTs were finally performed. (Test method: X2, G* Power 3.1.9.4, Franz Faul, University of Kiel, Kiel, Germany).

## Results

Out of 100 CBCT, 412 first and second mandibular molars were present in the CBCT images, which 206 were mandibular first permanent molars and 148 fulfilled the inclusion criteria.

Out of 148 mandibular first permanent molars, 49 had MMC. The prevalence of the MMC was 33,11%. Among the 49 canals identified, all of them had a separate orifice from the MB and ML canals (Fig. 1) and 16 were in male sex and 33 in female sex ( Table 1). There was no statistically significant difference between sex and prevalence of MMC (*P* < 0.05). It should be noted that the columns with the variable sex show the number of individuals of that sex, not the number of individuals with that trait.

The gender column showed the number of pieces of that gender, not the number of people with that trait.

The mean distance between the mesial canals was 1.76 ± 0.75 mm, males presented a higher distances (1.96 ± 0.14 mm) in comated with females (1.66 ± 0.09 mm) The same distance was examined into ranges; in the group 0-1 mm, there were 31 molars (20,95%), in the group 1-2mm there were 53 molars (35,81%), in the group 2-3mm there were 52 molars (35,14%), in the group 3-4mm there were 12 molars (8,11%), being the most common range was 1-2 mm.

Moreover, the ranges were divided according to gender; in men were found 9 molars in the group 0-1 (6,08) and 22 in females (14,86%), 10 molars in the men group 1-2 (6,76) and 43 molars in females (29,05%), 25 molars in the males group 2-3 (16,89%) and 27 molars in females (18,24%) and 5 molars in the males group 3-4 (3,38%) and 7 molars in females (4,73%). According to sex, the most common range in men were 2-3, and women 1-2 ( Table 1).

The distance between mesial canals was related to the presence of the MM canal. In the group 0-1mm, there was 1 molar with mesiocentral canal (0,68%); in the group 1-2mm, there were found 17 molars with mesiocentral canals (11,49%); in the group 2-3mm, there were found 25 molars with mesiocentral canals(16,89); and in the group 3-4mm, there were found 6 molars with mesiocental canals (4,05%). The most common range of distance between the mesial canals where the MM canal was localized was 2-3 mm, being statistically significant (*p*<0,05) ( Table 2).

The anatomical conFiguration of the mesial canals was studied according to Vertucci classification ( Table 3). The anatomical conFiguration not classifiable according to Vertucci was found in 23 pieces (23%) ( Table 3).

There was a significant difference between the presence of the mesiocentral canal and the distance between the mesial canals (*p*=0.001). The strength of the relationship was 0.38 (Cramers’ V). The presence of the mesiocentral canal was influenced by the distance between the MV and ML canals ( Table 4).

The relationship between the two variables, grouped by ranks, had a significant difference (*p*=0.001) with a relationship strength of 0.41 (Cramer’s V). The presence of the mesiocentral canals was influenced by the range of the distance between MV-ML canals ( Table 4).

There was no significant difference between sex and the presence of the mesiocentral canal by range (*p*=0.013) ( Table 4).

On the other hand, when it was observed that the relationship between the distance of the MV and ML canals and gender had a significant difference (*p*=0.05) with a strength of relationship of 0.320 (Cramer’s V) ( Table 4).

## Discussion

The mesial roots of mandibular first molars display a great variation in canal configuration (19). There is great variability in the prevalence of middle mesial canal in the permanent mandibular first molar (9) (4,10,14-16,20-24) and limited data in this regard in the Spanish subpopulation. This is the first study to analyse the prevalence of mesiocentral canal in the permanent mandibular first molar in a sample of Spanish patients and to identify factors associated with this prevalence.

Considerable importance has been gained by CBCT imaging in endodontics, including anatomical variations such as mesiocentral canals. Patel et Horner discussed about the importance of CBCT imaging in the diagnosis of endodontic treatment. They underlined the radiation dose was kept As Low As Reasonably Achievable (25). Furthermore, further studies were needed to asses the use of CBCT for diagnosis and management of endodontic problem (26). Recently, the European Society Of Endodontology published an article which positioned statement for the use of CBCT in Endodontics (27).

Vertical partitions inside the root are resulted from the secondary dentin apposition occurs during tooth development. This process forms a third root canal in mandibular molars (Bhargav K, Sirisha K, Jyothi M, Boddeda MR. Endodontic management of contralateral mandibular first molars with six root canals. J Conserv Dent. 2017;20:282-5). The results of our study showed that the prevalence of middle messial canals were 30,82%. This is higher as compared with other studies. The use of CBCT as a diagnosis method might be the cause of this hight results. (9) (2,4,24,10,14-16,20-23).

It is interesting how the percentages vary from study to study. This could be due to the different methods used, especially CBCT, where the results vary depending on the resolution. On the other hand, the most striking data published so far, which no study comes close to, is the prevalence obtained by Azim *et al*. in their study. All published articles refer to this because of its high percentage. This may also be due to the fact that they performed a study with CBCT and microscopy over a relatively low age range. These are two factors that may change the results obtained, as they show in their study that both factors are related (14). However, in the article published by Al-Maswary *et al*. there was no relationship between age and the presence of the mesiocentral canal (24). Akbarzadeh *et al*. talked about there was a great deal of controversy regarding this relationship, as there are very conflicting studies (22).

Regarding the use of the microscope, Tahmasbi *et al*. argued that the results obtained in the study were due to the fact that they count all the isthmuses found with the microscope as mesiocentral canal and they did not correspond to a real third canal. On the other hand, in the same study, Azim *et al*. explained this high percentage by the fact that the study was carried out in a very young age group in which the calcification processes have not yet occurred. Furthermore, Tahmasbi *et al*. stated that if they add up the prevalence of the mesiocentral canal and the isthmus in the apical third, they obtain a percentage of 53.3%, very similar to that obtained by Azim *et al*. which explains the high rate of endodontic failure and the importance of its detection for success.

The mean mesial canal distance found was 1.76 +- 0.75mm. This was quite different from other studies (2,9,20,22,28).

It was studied the relationship between the distance between the mesial canals to the presence of the mesiocentral canal. It was found that the greater the distance between the canals, the greater the probability of finding this third mesial canal, which was 50% in the range of distance 3-4.

Some studies are agree that the greater the interorifice distance, the greater the probability of finding the mesiocentral canal, stating that when the distance is 3-4mm, there is a 50% probability of finding the mesiocentral canal (9,20). Other authors found opposite resutls in their studies (22).

In terms of gender, there was no significant difference between gender and the presence of the mesiocentral canals. This is according to other previous studies (24). However, it was observed that distance was an influential factor in the presence of middle mesial canals. It was found that there was a significant difference between sex and the distance between mesial canals. The same result was found between sex and the range of the distance of mesial canals. Some authors discussed this in previous studies (9).

There was no significant difference between the presence of a mesiocentral canal and the second distal canal. In the review by Bansal *et al*., they found that there may be a possible relationship between the presence of the mesiocentral canal and the distolingual canal, being more likely to be found when the DL is present, although on the other hand they also find studies that say there is no relationship (28). The same was suggested by Penukonda *et al*., where they found several studies stating that when a distolingual canal is present, a mesiocentral canal is more likely to be found (2).

## Figures and Tables

**Table 1:** Distance between mesial canals of lower molars, grouped by rank and disaggregated by sex as well as the total mesiocentral canal presence and its difference by sex.

Molars n=148			Sex			
	Total		Men n=33		Women n=67	
MV-ML	X±(SD)^ €^	IC_95_^€^	X±(SD)^ ϒ^	IC_95_^ϒ^	X±(SD)^ ϒ^	IC_95_^ϒ^
	1,76± 0,08	1,61- 1.91	1,96 ±0,14	1,68-2,23	1,66 ± 0,09	1,48-1-83
Range MV - ML	n^$^	%^$^	n^&^	%^&^	n^&^	%^&^
0-1 mm	31	20,95	9	6,08	22	14,86
1-2 mm	53	35,81	10	6,76	43	29,05
2-3 mm	52	35,14	25	16,89	27	18,24
3-4 mm	12	8,11	5	3,38	7	4,73
	n	%	n^$^	%^$^	n^$^	%^$^
Presence MM	49	33,11	16	10,81	33	22,29

Note: €MV-ML= Mean distance between the mesial canals; ϒMV-ML= Mean distance between mesial canals according to sex; $Range MV-ML= range of mesial canal distances showing how many molars have this distance between their mesial canals. &Distance ranges by sex; àPrevalence of MM canal in mandibular molar by sex. MM = middle mesial canal, mesiocentral canal. MV= mesiovestibular; ML= mesiolingual; mm=millimetres.

**Table 2:** Relationship between the presence of the mesiocentral canal and the distance between the mesial canals grouped by rank.

Molars n= 148	Range MV- ML	Presence of mesiocentral canal (MM)			
		n		%	
	Yes	No	Yes	No
0-1 mm	1	30	0,68	20,27
1-2 mm	17	36	11,49	24,32
2-3 mm	25	27	16,89	18,24
3-4 mm	6	6	4,05	4,05

Note: n= Number of molars with or without mesiocentral canal in the range. %) Percentage corresponding to the value of n. MM = middle mesial canal, mesiocentral canal; MV= mesiovestibular; ML= mesiolingual; mm=millimetres.

**Table 3:** Vertucci type II and unclassifiable configuration, grouped according to sex.

Molars n= 159 *	Total, n (%)	
^£^Vertucci configuration II	106	66,66
Non-classifiable configuration	53	33,33
Vertucci configuration II	Non-classifiable configuration
^¥^Sex n (%) N=100	77 (77%)	23 (23%)
Men n=33	27	6
Women n=67	50	17

*Number of molars where was studied this anatonimal configuration. £ Number and percentage of molars with this anatomical configuration. ;¥ Same values as A, but grouped by sex.

**Table 4:** Anatomical relationships between the different measurements as well as their analysis with respect to the presence of radix entomolaris and the sex of the participant.

Molars n=148
	Presence of mesiocentral canal	Presence of distolingual canal
MV-ML	p=0.001*** / Cramer's V=0.38	p=0.460 /Cramer's V=0.65
DV-Cortical	p=0.100 / Cramer's V=1.01	---
Range MV-ML	p=0.001*** / Cramer's V= 0.41	---
	Sex	Radix
Range MV-ML	p=0.01** / Cramer's V=0.27	---
MV-ML	p=0.05* / Cramer's V=0.32	---
Molars n=148		
Presence of mesiocentral canal	p=0.607 / Cramer's V=0.05	---

*P*-value and Cramer’s V relationships between the different variables. Significance set at *p*< 0.05. ** *p*< 0.01; *** *p*< 0.001.

## Data Availability

The datasets used and/or analyzed during the current study are available from the corresponding author.
